# Amorphous nitride semiconductors with highly tunable optical and electronic properties: the benefits of disorder in Ca–Zn–N thin films†

**DOI:** 10.1039/d4mh01525h

**Published:** 2024-12-16

**Authors:** Elise Sirotti, Stefan Böhm, Gabriel Grötzner, Maximilian Christis, Laura I. Wagner, Lukas Wolz, Frans Munnik, Johanna Eichhorn, Martin Stutzmann, Verena Streibel, Ian D. Sharp

**Affiliations:** a Walter Schottky Institute, Technical University of Munich 85748 Garching Germany sharp@wsi.tum.de; b Physics Department, TUM School of Natural Sciences, Technical University of Munich 85748 Garching Germany; c Helmholtz-Zentrum Dresden-Rossendorf 01328 Dresden Germany

## Abstract

Semiconducting ternary nitrides are a promising class of materials that have received increasing attention in recent years, but often show high free electron concentrations due to the low defect formation energies of nitrogen vacancies and substitutional oxygen, leading to degenerate n-type doping. To achieve non-degenerate behavior, we now investigate a family of amorphous calcium–zinc nitride (Ca–Zn–N) thin films. By adjusting the metal cation ratios, we demonstrate band gap tunability between 1.4 and 2.0 eV and control over the charge carrier concentration across six orders of magnitude, all while maintaining high mobilities between 5 and 70 cm^2^ V^−1^ s^−1^. The combination of favorable electronic properties, low synthesis temperatures, and earth-abundant elements makes amorphous Ca–Zn–N highly promising for future sustainable electronics. Moreover, the successful synthesis of such materials, as well as their broad optical and electrical tunability, paves the way for a new class of tailored functional materials: amorphous nitride semiconductors – ANSs.

## Abstract 2

In amorphous Ca–Zn–N, charge carrier concentrations can be tuned across six orders of magnitude, without compromising their high mobilities and while offering tunable band gaps in the range from 1.4 to 2.0 eV, by solely adjusting the Ca to Zn ratio.

## Graphical abstract

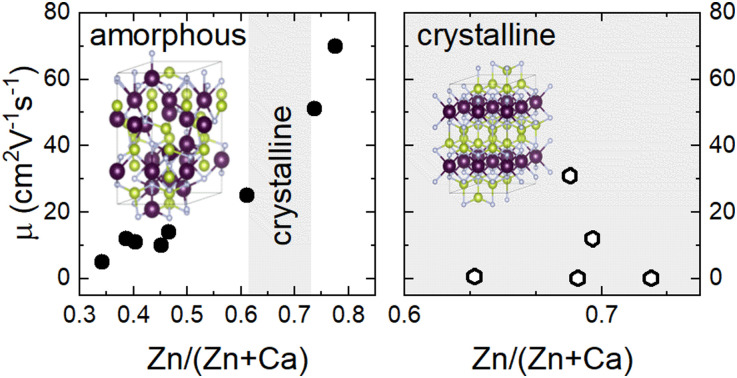

**New concepts**

This research investigates the previously unexplored class of amorphous nitride semiconductor materials, demonstrating that they provide highly controllable optoelectronic properties that are competitive with those of advanced amorphous oxide semiconductors. By comparing the physical characteristics of both crystalline and amorphous calcium–zinc nitride (a-Ca–Zn–N) thin films, we show that disorder offers specific benefits for enabling broadly tailored physical characteristics. By adjusting the Ca to Zn ratio, charge carrier concentrations can be tuned across six orders of magnitude, without compromising their high mobilities. Moreover, these materials offer tunable band gaps in the range from 1.4 to 2.0 eV. Our findings reveal that a-Ca–Zn–N can be synthesized at low temperatures using earth-abundant elements, a significant advantage for realizing sustainable, cost-effective electronics. Looking forward, amorphous nitride semiconductors such as a-Ca–Zn–N offer new opportunities for transformative impact in optoelectronics applications, including for solar energy harvesting, thin film electronics, and display technologies.

## 1. Introduction

Ternary nitrides have received increased interest in recent years due to their promising optoelectronic properties,^1^ which are suitable for a range of applications, including solar energy conversion,^2^ power electronics,^3^ and light-emitting diodes.^4^ However, the defect chemistry of nitride materials, including the low formation energies of intrinsic defects, cation disorder, and poorly controlled incorporation of impurity atoms, hinders the development of otherwise promising compounds for industrial applications.^5^ In the past, oxide-based semiconductors showed similar problems, which were partially addressed through the development of amorphous oxide semiconductors (AOSs). A prime example AOS is amorphous indium–gallium–zinc oxide (a-IGZO), which is now extensively used in display technologies and thin film transistors.^6,7^ Contrary to intuition, AOSs show attractive electrical properties^6–8^ due to their large area uniformity and absence of grain boundaries, which are often responsible for low mobilities in crystalline materials.^9,10^ Moreover, AOSs show chemical bonding properties that lead to favorable electron transport characteristics even if no long-range order exists in the material. In an AOS, the conduction band minimum (CBM) mainly consists of spherical s-orbitals of large atoms, facilitating orbital overlap and reducing sensitivity to angular variations to the next atomic neighbor.^11^ This orbital overlap with next-neighbor atoms leads to a strong hybridization of the CBM, which is responsible for the high mobilities reported in literature.

A materials family closely related to AOSs are amorphous oxynitride semiconductors (AONSs), which exhibit good electrical and tunable optical properties that can be varied by modifying the oxygen-to-nitrogen anion ratios. The oxygen content in AONS systems typically exceeds 30 at%, with crystallite nitride nucleation occurring for lower oxygen contents. Extensively studied AONS systems include indium–gallium–zinc oxynitride (a-IGZON),^12^ indium–tin oxynitride (a-ITON),^13^ tin oxynitride (a-SnON),^14^ indium–zinc oxynitride (a-IZON),^15^ tantalum oxynitride (a-TaON),^16^ and zinc oxynitride (a-ZnON).^17–21^ Among these examples, a-ZnON shows the highest mobilities. However, the charge carrier concentration of a-ZnON is also very high and difficult to tune below 10^18^ cm^−3^. Although it has been shown that Mg doping of a-ZnON can help decrease the charge carrier concentration to 10^15^ cm^−3^, this decrease comes at the cost of considerably reduced charge carrier mobilities.^20^ Hence, the range of practical applications remains limited for this material.

In contrast to oxides and oxynitrides, literature reporting the synthesis and (opto)electronic properties of amorphous nitride semiconductors (ANSs) is particularly scarce. In fact, most literature on ANSs focuses on their application as non-semiconducting amorphous coatings for increased temperature and oxidation resistance,^22–24^ diffusion barriers,^25^ electrical insulators,^26^ surface passivation layers for silicon solar cells,^27^ or as components of MEMS devices.^28^ Explored material systems include (TM, Si)N where TM = Ti, Zr, W, Mo, or Ta,^22,23,25^ for amorphous coatings, as well as amorphous nitrides of (Ta, Mo) N,^29,30^ CrCuTi(B, N),^31^ and (Al, TM)N with TM = Ti or Cr^32^ as diffusion barriers. To the best of our knowledge, there exists only one report of an ANS for electronics applications: in 2015, Fujioka filed a patent for an InGaAlN-based thin film transistor, reporting equivalent electrical characteristics for monocrystalline, polycrystalline, and amorphous InGaAlN channel layers.^33,34^ Hence, amorphous nitride semiconductors remain a largely unexplored field of research.

Within this context, the calcium–zinc nitride (Ca–Zn–N) material system is particularly intriguing. While Ca–Zn–N has already been reported in two different crystalline compositions, CaZn_2_N_2_ and Ca_2_ZnN_2_, reports on amorphous alloy films are lacking to the best of our knowledge. For crystalline CaZn_2_N_2_ and Ca_2_ZnN_2_, theoretical studies predict a direct band gap of 1.8 eV and an indirect band gap of 1.62 eV, respectively.^35^ Moreover, small electron and hole effective masses are reported for both stoichiometries.^35^ Density of states (DOS) calculations show that in CaZn_2_N_2_, Ca 4s-orbitals mainly contribute to the CBM, whereas for Ca_2_ZnN_2_, the CBM is dominated by Ca 3d and N 2p states.^35,36^ Besides the different natures of their CBMs, it has been reported that both phases can be doped both n- and p-type. In both phases, the main n-type dopants are nitrogen vacancies (*v*_N_), showing a relatively low formation energy,^35,37^ while the main p-type dopants are Na and K, acting as acceptors on the Ca site.^35^

Crystalline Ca–Zn–N configurations, c-CaZn_2_N_2_ and c-Ca_2_ZnN_2_, have been synthesized successfully in powder form by high-pressure synthesis.^35,38^ In addition, the Zn-rich c-CaZn_2_N_2_ phase has been grown in thin-film form by molecular beam epitaxy.^39^ For c-Ca_2_ZnN_2_, Hinuma *et al.* reported an indirect band gap of ∼1.6 eV and a direct band gap of 1.9 eV, in good agreement with theory.^35^ For c-CaZn_2_N_2_ in powder form, which included a Zn impurity phase, Hinuma *et al.* reported an absorption threshold of 1.9 eV at 300 K and red photoluminescence from band-to-band transitions.^35^ For epitaxial c-CaZn_2_N_2_ on YSZ(111) and GaN(0001), as well as polycrystalline Ca_2_ZnN_2_ on silica glass, Tsuji *et al.*^39^ reported band gaps between 1.93 and 2.04 eV. The band gaps can be further tuned between 1.8 and 3.2 eV by substituting Mg for Zn,^40^ as already had been hypothesized by Hinuma *et al.*^35^ Bare c-CaZn_2_N_2_ films grown epitaxially on YSZ(111) show p-type conductivity with very low charge carrier concentration in the range of (7–70) × 10^12^ cm^−3^, mobilities of 0.1–1 cm^2^ V^−1^ s^−1^, and large resistivities on the order of 10^6^ Ω cm.^39^ In contrast, polycrystalline c-CaZn_2_N_2_ films grown on silica glass show n-type conductivity with carrier densities in the range of (5–10) × 10^13^ cm^−3^, mobilities of 3–6 cm^2^ V^−1^ s^−1^, and resistivities of 2 × 10^4^ Ω cm.^39^

Despite these promising results, the mobilities remain low compared to theoretical predictions, most likely due to increased electron scattering at grain boundaries, which are also present and become even more pronounced in previously reported epitaxial films.^39^ In addition, the more Ca-rich phase, Ca_2_ZnN_2_, has also been reported experimentally in crystalline powder form, but to the best of our knowledge, no electrical measurements have been reported. Overall, the tunability of the band gap over a large part of the visible spectrum, the small electron and hole effective masses predicted by theory, the ability of these materials to be controllably doped over a broad range, and the spherical s-orbital dominating the CBM of CaZn_2_N_2_ raised our interest in investigating amorphous and crystalline compounds composed of various concentrations of Ca, Zn, and N.

In the present work, we use a plasma-assisted molecular beam epitaxy (PA-MBE) system to grow Ca–Zn–N thin films with varying Ca and Zn contents. Energy dispersive X-ray spectroscopy (EDX) combined with X-ray diffraction (XRD) analysis reveals that there exists only a small compositional window in which crystalline thin films in the CaZn_2_N_2_ structure emerge, while off-stoichiometric compositions lead to amorphous Ca–Zn–N thin films. Photothermal deflection spectroscopy (PDS) demonstrates that within our family of amorphous thin films, the band gap can be tuned between ∼1.4 eV and ∼2.0 eV by varying the Zn-to-Ca ratio. Focusing on the amorphous Ca–Zn–N films, we show that the elemental composition strongly influences the electrical transport properties. Importantly, the resistivity and charge carrier concentrations of the a-Ca–Zn–N thin films can be adjusted over six orders of magnitude without negatively impacting the Hall mobility. The insights gained in the present work highlight the advantage of amorphous Ca–Zn–N over crystalline CaZn_2_N_2_ and indicate that such amorphous nitride semiconductors, characterized by low processing temperatures and earth-abundant elements, can serve as highly tunable materials for sustainable thin film electronics applications.

## 2. Results and discussion

### 2.1 Thin film synthesis and composition

Ca–Zn–N thin films were deposited by PA-MBE in an ultra-high vacuum environment (see Experimental section for details). In brief, the Zn and Ca contents of the thin films were controlled by adjusting the temperatures of the Ca and Zn effusion cells and by varying the substrate temperature between 150 °C and 250 °C. The N_2_ flux supplied to the plasma cell was kept constant and set to 0.5 sccm or 0.8 sccm. Various substrates were used in initial trials to achieve epitaxial CaZn_2_N_2_ (see Table S1 for detailed deposition conditions, ESI†), which turned out to be challenging due to reasons mentioned later in this work. When exposed to atmospheric conditions, Ca-rich samples oxidized within seconds. To prevent oxidation upon air exposure, a GaN capping layer was deposited on all synthesized Ca–Zn–N films without breaking vacuum. In a previous study, we already reported the successful application of GaN capping layers to effectively protect Zn_3_N_2_ thin films for periods of years.^41^

The Ca and Zn content of all films was determined by EDX, while the nitrogen and oxygen contents in the films were characterized in detail by elastic recoil detection analysis (ERDA). Spatial EDX mapping up to a magnification of 15 000× reveals homogeneous elemental profiles and no indications for compositional clustering. ERDA indicates a variation in the oxygen content between 1–8 at%. Although the oxygen content decreases linearly with increasing nitrogen content, it shows no linear correlation with either the metal ratios or the growth rate (Fig. S1, ESI†). However, the ERDA results suggest an increase in oxygen content for Ca contents >30 at%. In the following, the Ca–Zn–N films are labeled according to their Zn/(Zn + Ca) ratios, referred to as “Ca–Zn–N(ratio)”.

### 2.2 Structural properties

**Fig. 1:**
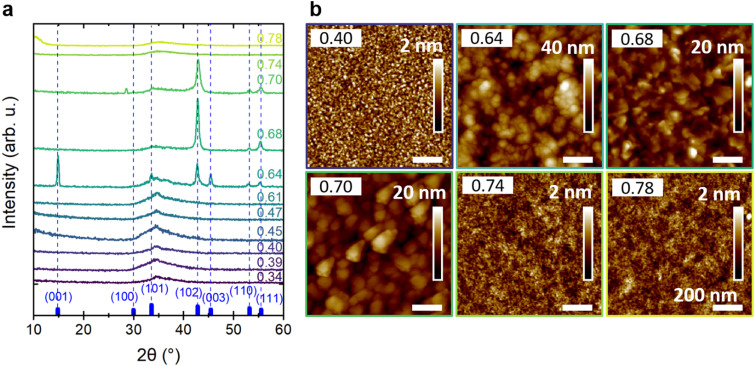
Structure and morphology of Ca–Zn–N thin films as a function of increasing Zn/(Zn + Ca) ratios. (a) X-ray diffraction patterns of the Ca–Zn–N thin films for Zn/(Zn + Ca) ratios between 0.34 and 0.78 (blue-green), as indicated on the right side of the panel. The reference diffraction pattern generated with VESTA^42^ for crystalline CaZn_2_N_2_ (mp-1029258)^43^ and the corresponding planes are shown in blue. (b) Corresponding atomic force microscopy images of Ca–Zn–N thin films. The in-plane scale bar of 200 nm applies to all images. The vertical roughness scale bar starts at 0 nm in all cases.

Fig. 1a shows the X-ray diffraction patterns of Ca–Zn–N thin films with increasing Zn/(Zn + Ca) ratios from 0.34 to 0.78. The limits are selected according to the ideal metal atomic ratios of 0.33 and 0.67 for Ca_2_ZnN_2_ and CaZn_2_N_2_, respectively. Interestingly, films with metal ratios within the range of 0.34–0.61 and 0.74–0.78 show only a broad diffuse reflection between 30° and 40°, indicating an amorphous structure lacking long-range order. The reported amorphous samples were grown on AlN, Al_2_O_3_, YSZ, and MgO substrates. Crystallization of such films with off-stoichiometric metal ratios by post-deposition annealing in nitrogen and vacuum (not shown here) was not successful, independent of the substrate used. At temperatures as low as 350 °C, Zn begins to cluster and diffuse out of the films, limiting the available temperature range for crystallization. From the 11 samples reported here, only Ca–Zn–N(0.64) on YSZ, Ca–Zn–N(0.68) on GaN, and Ca–Zn–N(0.70) on Al_2_O_3_ show reflections at 2*θ* angles corresponding to the expected diffraction pattern of CaZn_2_N_2_ (Fig. 1), *i.e.*, reflections at 42.8°, 53.2°, and 55.5° of the (102), (110), and (111) planes, respectively. The Ca–Zn–N(0.64) film shows two additional reflections at 14.8° and 45.4°, corresponding to the diffraction from (001) and (003) planes, respectively, and one reflection with small intensity at 33.5°, corresponding to the (101) plane. According to the VESTA^42^-generated XRD pattern of CaZn_2_N_2_ based on the crystallographic file (.cif) mp-1029258 from the Materials Project,^43^ the reflection at 33.5° should have the highest intensity. The low intensity or even absence of this reflection, which is also not observed in the rocking curve measurements, hints at textured Ca–Zn–N(0.64), Ca–Zn–N(0.68), and Ca–Zn–N(0.70) thin films. For Ca–Zn–N(0.70), a low-intensity reflection at 28.5° could not be assigned unambiguously, but could potentially correspond to the (311) diffraction of Ca_2_O_3_.

Additionally, atomic force microscopy measurements clearly reveal changes in morphology for different Zn/(Zn + Ca) ratios (Fig. 1b). Specifically, the amorphous samples (a-Ca–Zn–N) are characterized by smooth surfaces with an RMS roughness of only 0.33 nm, 0.24 nm, and 0.21 nm for Ca–Zn–N(0.40) on Al_2_O_3_, Ca–Zn–N(0.74) on AlN, and Ca–Zn–N(0.78) on AlN, respectively. These results are consistent with the absence of crystallinity when the elemental ratio deviates too far from the ideal stoichiometry of the crystalline compound. In contrast, the c-CaZn_2_N_2_ thin films are dominated by large grains with an RMS roughness of 6.4 nm, 2.3 nm, and 2.2 nm for Ca–Zn–N(0.64), Ca–Zn–N(0.68), and Ca–Zn–N(0.70), respectively. In detail, Ca–Zn–N(0.64), grown on YSZ(100), shows spherical grains with a size of ∼50 nm, while Ca–Zn–N(0.68), grown on GaN(0001), is characterized by larger grains with a flaked structure. The latter agrees with the morphology reported by Tsuji *et al.* for CaZn_2_N_2_ grown on silica glass.^39^ Ca–Zn–N(0.70), grown on Al_2_O_3_(0001), shows triangular grains stacked on each other, all oriented with longer facets in the same direction. The triangular grains are ∼180 nm wide and ∼130 nm long. Tsuji *et al.* observed a similar triangular grain shape for CaZn_2_N_2_ films grown epitaxially on YSZ(111).^39^

**Fig. 2:**
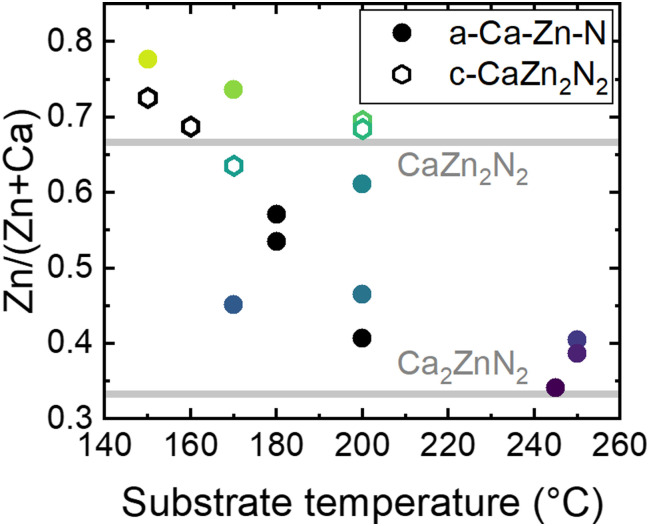
Metal ratio of amorphous (a-Ca–Zn–N, circles) and crystalline (c-CaZn_2_N_2_, hexagons) thin films determined by EDX as a function of the substrate temperature. The colored data points represent the thin films with corresponding XRD patterns shown in Fig. 1. Horizontal grey lines at the expected stoichiometric ratios of Ca_2_ZnN_2_ and CaZn_2_N_2_ are added as a guide to the eye.

The fact that growth on YSZ(100) and Al_2_O_3_(0001) can lead to amorphous or crystalline layers, indicates little influence of the substrate on the crystallinity of the samples. To confirm that the formation of c-CaZn_2_N_2_ is mainly influenced by the metal ratios in a sample, Zn/(Zn + Ca) is plotted as a function of the growth temperature (Fig. 2). The colored points represent the thin films from which the XRD patterns are reported in Fig. 1, and the black dots are additional samples grown during our study. For a substrate temperature <200 °C, the metal ratio can be tuned over a broad range by changing the temperature of the Zn and Ca effusion cells. Interestingly, independent of substrate temperature, we confirm the growth of crystalline CaZn_2_N_2_ for metal ratios between 0.64–0.73 and amorphous films for stoichiometries farther away from the ideal Zn/(Zn + Ca) = 0.67. For a substrate temperature >200 °C, we encountered great difficulty in increasing the Zn/(Zn + Ca) ratio to above 0.45, even at Zn effusion cell temperatures as high as 230 °C, due to the volatility of Zn in this temperature range (Fig. S2, ESI†). When the substrate temperature and Ca-to-Zn flux ratios are increased further, metallic Ca and Ca_2_N_3_ phases form (not further discussed here).

In the Zn-rich composition region, when initially targeting the growth of epitaxial c-CaZn_2_N_2_, we tested different types of substrates (YSZ(100), MgO(100), Al_2_O_3_(0001), GaN(0001), AlN(0001)) to potentially enhance crystallization. However, through our studies, we found that it is primarly the strong dependence of Zn incorporation on substrate temperature that dominates the growth of c-CaZn_2_N_2_, since the elemental composition window for crystalline growth is small. We did not succeed in growing epitaxial Ca_2_ZnN_2_. Indeed, in the Ca-rich composition region, independent of deposition temperature, all thin films with metal ratios close to the Ca_2_ZnN_2_ stoichiometry were amorphous, and no sample with the Ca_2_ZnN_2_ crystalline phase could be grown.

### 2.3 Optical properties

**Fig. 3:**
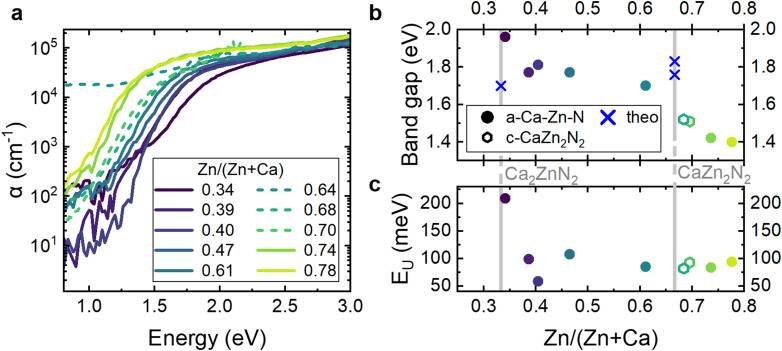
Optical properties of the Ca–Zn–N thin films with different Zn/(Zn + Ca) ratios. Dotted lines and empty hexagons represent crystalline samples (c-CaZn_2_N_2_). Solid lines and circles represent amorphous samples (a-Ca–Zn–N). (a) Photothermal deflection spectroscopy measurements of the absorption coefficient as a function of photon energy. (b) Band gap and (c) Urbach energies of the Ca–Zn–N thin films as a function of increasing Zn/(Zn + Ca) ratios. The band gap was determined with the Tauc plot method for direct band gap semiconductors (see fits in Fig. S3, ESI†). Additionally, the expected theoretical crystalline phase band gap values from the literature are provided as blue crosses.^35,36^

Analysis of absorption characteristics provides information on not only the optical properties but also on the structural disorder within semiconductors. Here, we evaluate the influence of chemical composition on the optical band gap and Urbach tail of the Ca–Zn–N thin films by photothermal deflection spectroscopy (PDS) (Fig. 3). In Fig. 3a, the absorption coefficient *α* is reported as a function of photon energy for the amorphous (solid lines) and crystalline (dotted lines) films. We observe a strong absorption edge with above band gap *α* > 10^5^ cm^−1^ for all Ca–Zn–N films. Moreover, the overall trend shows increasing absorption strength with increasing Zn content in the band-to-band absorption regime (*e.g.* above 2.0 eV). The absorption onset shifts to lower energies with increasing Zn content and was quantitatively determined with the Tauc plot method (Fig. 3b, fits in Fig. S3, ESI†). This analysis shows a decrease in the band gap energy from 2.0 eV to 1.4 eV with increasing Zn content. The band gap showed smaller values when determined with the Iso_04_ method, which is often used in literature for amorphous Si^44^ and is defined as the energy for which *α* is equal to 10^4^ cm^−1^ (Fig. S4, ESI†). Based on this Iso_04_ analysis, the band gap decreases linearly by ∼0.6 eV with increasing Zn content, from 1.8 eV to 1.2 eV.

To obtain information concerning defect states within the band gap, as well as to assess the possibility of free carrier absorption, we now consider the sub-band gap absorption characteristics. For Ca–Zn–N(0.34), the sub-band gap absorption is rather high, while for Ca–Zn–N(0.39) it decreases by one order of magnitude. With further increasing Zn content, the sub-band gap absorption also increases. Fitting an exponential function to the Urbach tail enables us to quantify the impact of structural disorder in our thin films (Fig. 3c). The obtained Urbach energies (Fig. 3c) show remarkably small values between 60 meV and 100 meV, with the exception of the Ca-rich sample Ca–Zn–N(0.34), which shows a high Urbach energy of ∼210 meV. These small Urbach energies are very promising, especially for a material that has not yet been optimized, suggesting a favorable defect tolerance, while there remains a large space for improvement through post-annealing processes and tuning of deposition parameters that influence disorder. For comparison, the intensively studied AOSs show even larger Urbach energies, including ∼150 meV for a-IGZO,^45^ between 160 meV to 210 meV for In_2_O_3_–SnO_2_,^46^ and 110 meV to 140 meV for zinc tin oxide (a-ZTO).^47^ For photovoltaic applications, intensively developed and optimized a-Si:H and a-CIGS film show Urbach energies below 50 meV.^47^

Interestingly, the crystalline films fit within the trend of a decreasing band gap with increasing Zn content observed for the amorphous films. Ca–Zn–N(0.68) with a metal ratio near the CaZn_2_N_2_ stoichiometry has a band gap energy of 1.5 eV, and Ca–Zn–N(0.70), which exhibits the best crystallinity on the basis of XRD characterization, also has a band gap of 1.5 eV. For the third crystalline sample, Ca–Zn–N(0.64), the strong sub-band gap absorption precludes quantitative determination of the band gap. In general, the observed values for c-CaZn_2_N_2_ are in line with theoretical predictions of optical band gaps of 1.8 eV^35^ and prior experimental reports of ∼2 eV for c-CaZn_2_N_2_ in thin film form.^39^ Overall, the significant change in sub-band gap absorption of crystalline films shows no trend with composition. This lack of a correlation may be attributed to the varying substrate temperatures, which were observed to impact the sub-band gap absorption of the films (not shown here). In particular, the sub-band gap absorption was observed to decrease with increasing growth temperature for the same film chemical composition, possibly due to the formation of metallic clusters at low growth temperature.

In summary, the absorption spectra of our Ca–Zn–N films with different stoichiometries show that the band gap increases linearly with increasing Zn content and is mainly influenced by composition but not by crystallinity. Moreover, the amorphous samples show low Urbach energies that are comparable to commercially relevant photovoltaic materials and are significantly smaller than those of intensively investigated AOs. This finding suggests a low concentration of electronically active structural defect states near the CBM or VBM, which, if present in high concentrations, could negatively impact the transport properties of the semiconductor films by shallow trapping.

### 2.4 Room temperature electrical properties

**Fig. 4:**
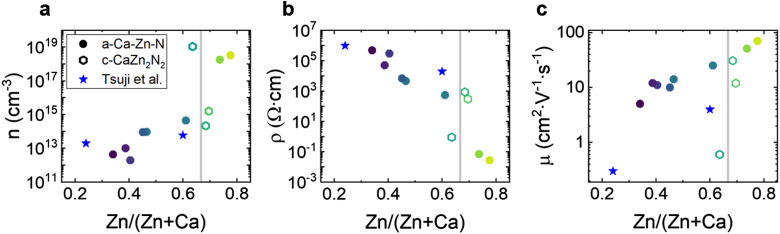
Electrical properties of the Ca–Zn–N thin films determined with room temperature Hall effect measurements. (a) Charge carrier concentration *n*, (b) resistivity *ρ*, and (c) mobility *μ* as a function of film composition. A vertical grey line corresponding to the stoichiometric ratio of CaZn_2_N_2_ is added as a guide to the eye.

To determine the influence of chemical composition on electrical transport properties of these tunable band gap compounds we first performed room temperature Hall measurements of the carrier concentration, resistivity, and mobility (Fig. 4). For all samples, irrespective of composition and structure, the Hall measurements indicate n-type conductivity.

For amorphous samples (filled points), the free charge carrier concentration increases by over six orders of magnitude, from 10^12^ cm^−3^ to 10^18^ cm^−3^, with increasing Zn content. Correspondingly, the resistivity of the amorphous samples decreases over seven orders of magnitude with increasing Zn content, from 4.9 × 10^5^ Ω cm for Ca–Zn–N(0.34) to 2.8 × 10^−2^ Ω cm for Ca–Zn–N(0.78). In addition, the Hall mobilities are remarkably large and increase with increasing Zn content, from 5 cm^2^ V^−1^ s^−1^ to 70 cm^2^ V^−1^ s^−1^. As expected, the sample with the largest Urbach energy (Ca–Zn–N(0.34), Fig. 3c), is characterized by the lowest mobility, which hints at the formation of electronically active defect states within the band gap. Compared to literature on AOSs used in commercial applications,^45^ the observed mobilities of these compounds are high, especially considering their comparatively low charge carrier concentrations. In the a-IGZO system, the highest mobility of 39 cm^2^ V^−1^ s^−1^ is achieved for amorphous Zn-doped In_2_O_3_.^48^ However, its free charge carrier concentration is 10^20^ cm^−3^, with no possibility to decrease it without negatively impacting the mobility.^48^ Similar to the case of AOSs, our amorphous nitride films exhibit increasing mobility with increasing charge carrier concentration. In summary, the high mobilities, along with the very low charge carrier concentrations of our a-Ca–Zn–N samples, are highly promising for optoelectronic applications, providing superior transport characteristics relative to many of the most actively developed amorphous oxide materials.

Interestingly, the crystalline samples (empty hexagons) show a trend opposite to the amorphous films, with a decrease in mobility with increased charge carrier concentration, most likely due to enhanced impurity scattering.^48^ The charge carrier concentration correlates with the sub-band gap absorption observed in Fig. 3a, showing an increased charge carrier concentration in the film with higher sub-band gap absorption. Two of the three crystalline samples reported here, Ca–Zn–N(0.68) and Ca–Zn–N(0.70), show promising mobilities of 12 and 31 cm^2^ V^−1^ s^−1^, which are higher than those reported for epitaxial CaZn_2_N_2_ thin films by Tsuji *et al.*^39^ The third crystalline film Ca–Zn–N(0.64), shows a mobility of only 0.6 cm^2^ V^−1^ s^−1^ and a high charge carrier concentration of 10^19^ cm^−3^. Additional samples grown at temperatures <170 °C with CaZn_2_N_2_ crystalline reflections detected by XRD also show very high charge carrier concentrations in the range of 10^21^ cm^−3^ (not shown here). The high charge carrier concentration of the films could arise from the formation of metallic clusters at low temperatures, even if not visible by XRD, or from an increased number of structural defects or grain boundaries. Indeed, Tsuji *et al.*^39^ hypothesized that their low mobilities come from the island growth mode of CaZn_2_N_2_, which results in the formation of thick grain boundaries and leads to large potential barriers between the grains, which are difficult for electrons to overcome. Overall, the observations on the superior electrical properties of a-Ca–Zn–N compared to c-CaZn_2_N_2_ films encouraged us to focus our subsequent investigations on amorphous rather than crystalline films. In particular, such ANSs, represent homogeneous and tunable materials system with low carrier concentrations and high mobilities.

### 2.5 Temperature-dependent electrical transport

**Fig. 5:**
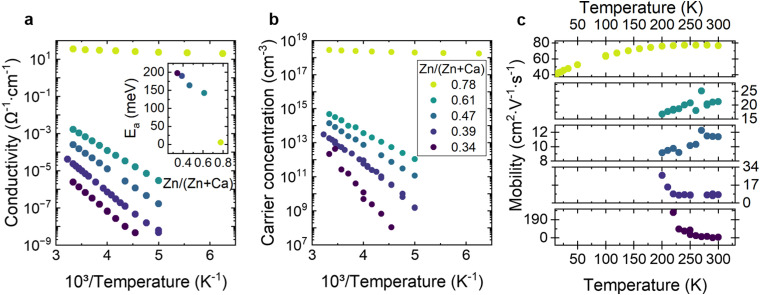
Temperature-dependent Hall effect measurements. (a) Conductivity and (b) charge carrier concentration as a function of inverse temperature. (inset in a) activation energy, determined with Arrhenius fitting of the temperature-dependent conductivity for increasing Zn/(Zn + Ca) ratios. (c) Hall mobility as a function of temperature for increasing Zn/(Zn + Ca) ratios. The legend in (b) applies to all figures.

In order to probe the electrical transport mechanisms and donor state energetics within this ANSs, we performed temperature-dependent Hall measurements for selected samples with different compositions (Fig. 5). While the measurements were performed between 10 K and 300 K, the results were unreliable below 200 K for most samples, most likely due to the formation of Schottky barriers at the semiconductor|metal contact interface. From these measurements, the temperature-dependent conductivity, charge carrier concentration, and mobility were quantified and analyzed.

As expected for non-degenerate semiconductors, the conductivity (Fig. 5a) and the charge carrier concentration (Fig. 5b) decrease exponentially with decreasing temperature. Based on these data, we determined the thermal activation energy *E*_a_ of the electrical conductivity (inset Fig. 5a). Analysis of the activation energy for charge transport is an effective method for identifying localized energy levels (trap states) and can be calculated by fitting the conductivity as a function of temperature using the Arrhenius equation.^49^ The activation energy determined from the freeze-out curve of the charge carrier concentration as a function of temperature shows identical ionization energies to the one determined by fitting the conductivity as a function of temperature. However, since the complete freeze-out curve could not be measured, these energies could represent either the donor ionization energy, or half of the donor ionization energy. Nevertheless, the samples with a metal ratio within 0.34–0.61 show activation energies that decrease from 198 meV to 143 meV with increasing Zn content. This observation follows the trend of the decreasing band gap with increasing Zn (Fig. 3a). Considering that the CBM is expected to comprise cation orbital states, this suggests that decreasing CBM energy leads to shallower donor states. Finally, the most Zn-rich sample, Ca–Zn–N(0.78), shows the lowest activation energy of 6 meV, indicating metallic conduction. This value is lower than the only reported activation energy on the Ca–Zn–N system, which was found to be ∼20 meV for the Ca-rich crystalline Ca_2_ZnN_2_ phase.^35^

The mobility (Fig. 5c) shows different temperature-dependent behaviors for different Zn contents. In the temperature range from 200 K to 300 K, Ca–Zn–N(0.34) and Ca–Zn–N(0.39) show a decrease in mobility with increasing temperature, characteristic of phonon scattering-limited conduction.^50^ By contrast, Ca–Zn–N(0.47) and Ca–Zn–N(0.61) show a linear increase in mobility with increasing temperature, indicating impurity scattering-limited conduction.^50^ Ca–Zn–N(0.78) shows increased mobility with increasing temperature in the 10 K to 260 K range, which then decreases between 260 K and 300 K. The observed peak of the mobility below room temperature (260 K) indicates a transition from defect scattering at low temperatures to phonon scattering at high temperatures for this sample.

Overall, the temperature-dependent conductivity measurement confirms semiconducting behavior for all a-Ca–Zn–N films. The investigated amorphous films show a decrease of the conductivity activation energy with decreasing band gap. Moreover, temperature-dependent mobility measurements suggest a change in the conduction behavior from phonon scattering-limited conduction in Ca-rich samples to ionized impurity scattering-limited conduction in Zn-rich samples.

## 3. Experimental section

### 3.1 Sample preparation

Ca–Zn–N thin films with different calcium to zinc ratios were grown with a Riber 32 molecular beam epitaxy system equipped with Zn and Ca effusion cells, and a radio frequency plasma source. The background pressure in the growth chamber increased from 10^−10^ mbar to 10^−9^ mbar after the introduction of Ca to the chamber. High-purity Ar (7.0 Ar, Linde) gas was supplied to the plasma source to ignite the plasma and was then replaced by high-purity N_2_ (7.0 N_2_, Linde) when the power of the plasma source reached 250 W. The N_2_ and Ar flows were provided by mass flow controllers and the N_2_ flux was varied between 0.5 sccm and 0.8 sccm. The temperature of the effusion cells was varied between 190–220 °C and between 400–410 °C for Zn and Ca, respectively. The substrate temperature was varied between 150 °C and 250 °C. Attempts to obtain epitaxial CaZn_2_N_2_, different substrates were used: Al_2_O_3_(0001) (Witec), AlN(0001) (Xiamen Powerway Advanced Material), Y-stabilized ZrO_2_ (YSZ)(100) (Alineason), and MgO(100) (MSE Supplies). Prior to growth, the substrates were immersed in a beaker with acetone and placed in an ultrasonic bath for 5 min. Then, the substrates were mechanically cleaned with a cotton swab and acetone, rinsed with acetone, and dried with nitrogen. Afterwards, the substrates were ultrasonicated in a beaker filled with isopropanol for 5 min. Finally, they were rinsed with isopropanol and dried with nitrogen. The substrates were then immediately loaded on the sample holder and stored in the load lock of the PA-MBE chamber. Before the start of growth, the substrate was additionally conditioned *via* N_2_ plasma exposure for 10 min. The list of deposited samples with their respective growth temperatures, growth times, and thicknesses can be found in Table S1 (ESI†).

### 3.2 Elemental characterization

Elemental characterization was performed using a Bruker XFlash6130 energy-dispersive X-ray (EDX) detector mounted on a Zeiss EVO MA10 scanning electron microscope (SEM) with an electron beam energy of 10 keV. The spot size on the sample was 1 μm in diameter. The resulting EDX spectra were quantified using the Phi(rho,z) method and the deconvolution function SeriesFit. The method showed consistent results for the Ca to Zn ratio but limited accuracy for the nitrogen and oxygen content due to the GaN capping layer on top of the sample and the varying contributions of N and O from the different underlying substrates. To remedy this shortcoming, depth-dependent elemental characterization was carried out by elastic recoil detection analysis (ERDA). ERDA were performed at the Helmholtz–Zentrum Dresden–Rossendorf (HZDR) using a 43 MeV Cl^7+^ ion beam. The angle between the sample normal and the incoming beam was 75° and the scattering angle was 30°. The analyzed area was ∼2 × 2 mm^2^. The recoil atoms and scattered ions were detected with a Bragg ionization chamber, which enables energy measurement and the identification of the atomic number, *Z*, of the elements within the film. Analysis of ERDA data was performed with the program Windf v9.3g.^51^

### 3.3 Structural characterization

For structural characterization, grazing incidence X-ray diffraction (GI-XRD) measurements were performed with a Rigaku SmartLab 3 kW system equipped with a Cu anode X-ray source and a HyPix-3000 detector. After sample surface alignment, the samples were measured at a fixed incident angle of *ω* = 0.5°, while 2*θ* was scanned from 10° to 60°. The scans were performed with 0.04° steps and a scan speed of 2° min^−1^. The reference.cif file (mp-1029258) for XRD pattern generation of CaZn_2_N_2_ using VESTA^42^ was retrieved from the Materials Project database version v2023.11.1.^43^ The film roughness and topology were determined using a Veeco MultiMode atomic force microscope (AFM) in tapping mode with NSG30 tips from TipsNano (tip height 14–16 μm, tip radius of curvature: 10 nm) and a Bruker Dimension Icon AFM in PeakForce mode (Quantitative Nanomechanics) with RFE SPA 75 tips with a nominal spring constant of 3 N m^−1^, operating at a forced frequency of 2 kHz.

### 3.4 Optical characterization

Optical absorption spectra were collected with a home-built photothermal deflection spectroscopy (PDS) setup. The sample was immersed in perfluorohexane (C_6_F_14_) in a quartz cuvette, which was placed on a translation and rotation stage for optical alignment, and was illuminated at normal incidence with focused monochromatic light obtained from a source containing Halogen and Xenon lamps (100 W Quartz Tungsten Halogen Lamp, G6.35 base and 75 W Short Arc Xenon lamp, respectively) passed through a monochromator (Bentham Instruments, TMc300). The incident light was chopped at 9 Hz. The absorption was probed using a HeNe laser (2 mW, 632.8 nm, HNL020L, Thorlabs) directed parallel to the sample surface, with deflection arising due to heat transfer from the absorbing sample and thermal lensing in the perfluorohexane. A 2D lateral sensing detector (PDP90A, Thorlabs) was used to track the deflected probe laser beam. The detector and the chopper were connected to a lock-in amplifier (SR830, Stanford Research Systems), which was used to isolate and amplify the signal at the modulation frequency. The measured sample thickness was used to determine the absorption coefficient from the measured PDS signal.

### 3.5 Electrical characterization

Hall measurements were performed using a LakeShore Model 8404 AC/DC Hall effect system operated in DC mode with a magnetic field of 0.9 T. For all samples, the high-resistance mode of the system was used (Keithley Model 6514 system electrometer). For the measurements performed at room temperature, indium foil was pressed onto the sample surface in the van der Pauw geometry and then clamped on a sample card with prober pins. The sample card was then inserted into the standard probe head of the LakeShore system. The temperature-dependent measurements were performed using a closed-cycle cryostat integrated into the LakeShore Model 8404 Hall system. However, the head of the cryostat in the variable temperature configuration required the sample to be glued to the sample holder. Silver wires were then used to connect the indium contacts in the van der Pauw geometry to the sample holder.

## 4. Conclusions

In conclusion, our findings shed light on the optical and electrical properties of amorphous ternary nitrides, revealing them to be a promising new class of materials for optoelectronic applications. To the best of our knowledge, Ca–Zn–N is the first reported amorphous nitride semiconductor offering properties that are well suited for use in thin film optoelectronic devices. By controlling the metal composition, we demonstrated the tunability of its optical band gap between 1.4 eV and 2.0 eV, making it a suitable solar absorber candidate. In addition, we showed that the resistivity and charge carrier concentration can be adjusted by over seven and six orders of magnitude, respectively, with electron mobilities ranging between 5 and 70 cm^2^ V^−1^ s^−1^, notably without exceeding charge carrier concentrations of 10^18^ cm^−3^. Its amorphous structure enables us to broadly control the concentration of free charge carriers in the material, primarily by adjusting the Ca to Zn ratio. Importantly, these amorphous Ca–Zn–N thin films are sustainable materials, exclusively composed of earth-abundant elements and synthesized at comparatively low temperatures, and possess properties that are highly competitive with – and even superior to – the intensively investigated and commercialized class of AOSs. Indeed, these ANSs feature smaller band gaps, reduced Urbach energies, and comparable mobilities at much lower carrier concentrations, thus opening pathways to a multitude of new applications. We expect that our findings could thus pave the way for a novel field of research targeting amorphous nitride semiconductors as tailored functional materials.

## Author contributions

E. S. conceptualization, data curation, formal analysis, investigation, project administration, validation, visualization and writing – original draft, S. B. investigation, G. G. investigation, M. C. validation, L. I. W. validation, L. W. investigation, F. M. investigation, formal analysis and validation, J. E. funding acquisition and writing – review & editing, M. S. conceptualization and writing – review & editing, V. S. writing – review & editing, I. D. S. supervision, funding acquisition and writing – review & editing.

## Data availability

The data supporting this article have been included as part of the ESI.†

## Conflicts of interest

There are no conflicts to declare.

## Supplementary Material

MH-012-D4MH01525H-s001

MH-012-D4MH01525H-s002
