# Supplementary material for: Amorphous nitride semiconductors with highly tunable optical and electronic properties: the benefits of disorder in Ca–Zn–N thin films
Source: Mater Horiz. 2024 Dec 16;12(6):1971–80. doi: 10.1039/d4mh01525h (PMC11667462; doi:10.1039/d4mh01525h)
Supplement: MH-012-D4MH01525H-s002 [file MH-012-D4MH01525H-s002.pdf]

Supplementary Information (SI) for Materials Horizons.
This journal is © The Royal Society of Chemistry 2024

Supporting information for

Amorphous nitride semiconductors with highly tunable optical and electronic properties: The benefits of disorder in Ca-Zn-N thin films

Elise Sirotti,[a,b] Stefan Böhm,[a,b] Gabriel Grötzner,[a,b] Maximilian Christis,[a,b] Laura I. Wagner,[a,b] Lukas Wolz,[b] Frans Munnik,[c] Johanna Eichhorn,[b] Martin Stutzmann,[a,b] Verena Streibel,[a,b] Ian D. Sharp [a,b]*

[1] Walter Schottky Institute, Technical University of Munich, 85748 Garching, Germany
[2] Physics Department, TUM School of Natural Sciences, Technical University of Munich, 85748 Garching, Germany
[3] Helmholtz-Zentrum Dresden-Rossendorf, 01328 Dresden, Germany
*Corresponding author e-mail: sharp@wsi.tum.de

Supplementary Table

Table S1: List of samples investigated in this work, including the metal ratios determined by EDX, the cation to anion ratio determined by ERDA, the growth conditions, and the sample thickness measured with a stylus profilometer. The growth conditions are described with the substrate temperature T_{Growth}, the growth time t_{Growth}, the nitrogen flux reaching the plasma cell powered at 400W N-flux, and T_{Zn} and T_{Ca}, the zinc and calcium effusion cell temperature, respectively. The expected ratios for c-$CaZn_2N_2$ are 0.67 and 1.5 for Zn/(Zn+Ca) and (Zn+Ca)/(O+N), respectively.

Zn/(Zn+Ca) (EDX)	(Zn+Ca)/(O+N) (ERDA)	Substrate	T_{Zn} (°C)	T_{Ca} (°C)	N-flux @400W	T_{Growth} (°C)	t_{Growth} (min)	Thickness (nm)
0.34	1.38	Al_2O_3 (0001)	220	400	0.5	245	180	125
0.39	1.41	Al_2O_3 (0001)	230	410	0.8	250	180	250
0.40	1.38	Al_2O_3 (0001)	220	400	0.8	250	180	170
0.41	-	AlN (0001)	200	400	0.5	200	120	230
0.45	-	MgO (100)	200	400	0.5	170	120	240
0.47	1.42	AlN (0001)	200	400	0.5	200	120	265
0.55	-	YSZ (100)	200	400	0.5	180	120	310
0.57	-	YSZ (100)	200	400	0.5	180	120	300
0.61	-	AlN (0001)	210	400	0.8	200	120	250
0.64	-	YSZ (100)	200	400	0.5	170	120	330
0.68	-	GaN:Fe (0001)	210	400	0.5	200	120	150
0.69	-	YSZ (100)	200	400	0.5	160	120	-
0.70	-	AlN (0001)	210	400	0.8	200	120	180
0.73	-	YSZ (100)	200	400	0.5	150	60	-
0.74	1.39	AlN (0001)	200	400	0.8	170	90	125
0.78	1.39	AlN (0001)	195	400	0.8	150	120	140

Supplementary Figures

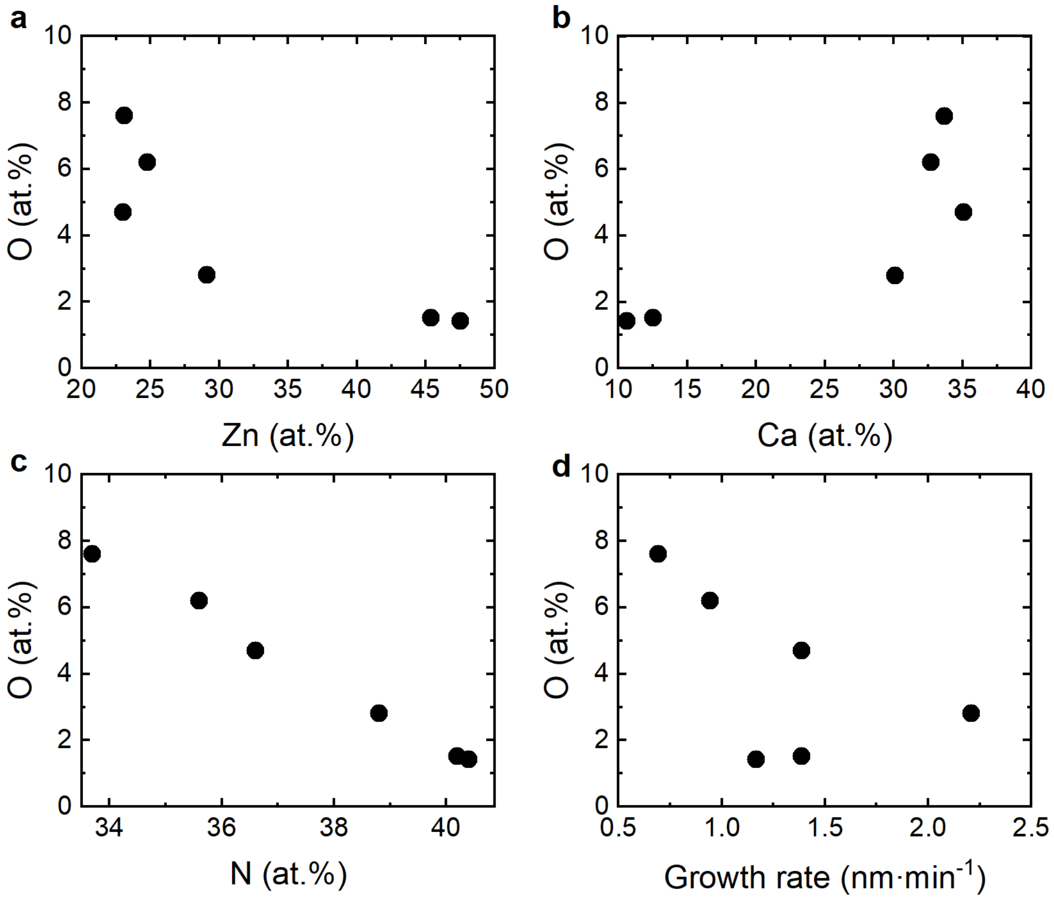

Figure S1. Oxygen content as a function of the concentration of the other elements, determined by ERDA (O and N) and RBS (Ca and Zn) measurements. The elemental composition is given in at.%. **a)** O as a function of Zn, **b)** O as a function of Ca. **c)** O as a function of N, and **d)** O as a function of the growth rate in nm·min^{-1}.

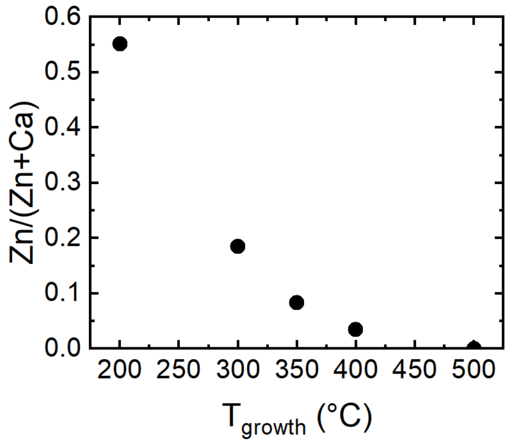

Figure S2. Zn to Ca ratio determined by EDX measurements as a function of the growth temperature. All growth parameters other than the substrate temperature were held constant, with

a Zn cell temperature T_{Zn} of 200 °C, Ca cell temperature of T_{Ca} of 450 °C, and N_2 flux of 0.8 sccm with a plasma power of 400 W.

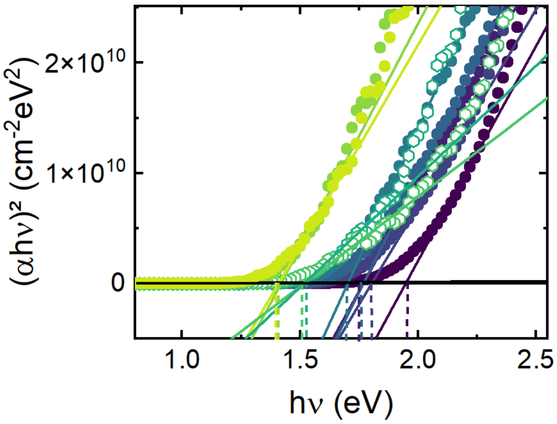

Zn/(Zn+Ca)	Band gap (eV)
0.78	1.40
0.74	1.42
0.70	1.51
0.68	1.52
0.61	1.70
0.47	1.77
0.40	1.81
0.39	1.77
0.34	1.96

Figure S3. Tauc plots analysis of the absorption coefficient of amorphous (filled circles) and crystalline (empty hexagons) Ca-Zn-N films as obtained by PDS (see Figure 3a for complete spectra). The table on the right-hand side provides the band gap values as a function of composition, as obtained from this analysis.

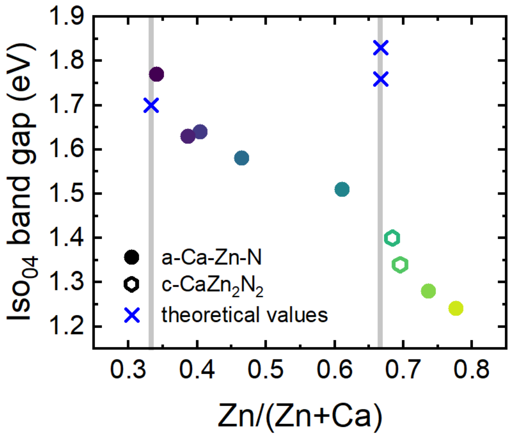

Figure S4. Band gap determined with the Iso_{04} method, defined as the energy for which α is equal to 10^4 cm^{-1}, determined for the amorphous and crystalline Ca-Zn-N films. Vertical grey lines at the expected stoichiometric ratios of Ca_2ZnN_2 and $CaZn_2N_2$ are added as a guide to the eye. Additionally, the expected theoretical crystalline phase band gap values from the literature are provided as blue crosses.[1, 2]

References

1. Hinuma, Y., Hatakeyama, T., Kumagai, Y., Burton, L.A., Sato, H., Muraba, Y., Iimura, S., Hiramatsu, H., Tanaka, I., and Hosono, H., et al. (2016). Discovery of earth-abundant nitride semiconductors by computational screening and high-pressure synthesis. Nature communications *7,* 11962.

2. Sajidul Islam, M., Ahmed, R., Mahamudujjaman, M., Islam, R.S., and Naqib, S.H. (2023). A comparative study of the structural, elastic, thermophysical, and optoelectronic properties of CaZn2X2 (X = N, P, As) semiconductors via ab-initio approach. Results in Physics *44,* 106214.
